# The Evolutionary Flexibility of the *Drosophila* Circadian Clock: Network Constraints or Adaptive Freedom?

**DOI:** 10.1093/gbe/evag061

**Published:** 2026-03-07

**Authors:** Leo Douglas Creasey, Petar Borisov Petrov, Eran Tauber

**Affiliations:** Department of Evolutionary and Environmental Biology, Institute of Evolution, University of Haifa, Haifa, Israel; Infotech Institute, University of Oulu, Oulu, Finland; Department of Evolutionary and Environmental Biology, Institute of Evolution, University of Haifa, Haifa, Israel

**Keywords:** gene networks, protein networks, *Drosophila melanogaster*, circadian clocks, pleiotropy, molecular co-evolution

## Abstract

The study of network evolution is critical to understanding how complex biological processes arise and adapt over time. Protein networks, composed of interacting components, can exhibit varying degrees of conservation and flexibility, enabling organisms to fine-tune their responses to environmental changes. Using the circadian clock system in *Drosophila* as a case study, we explore how such networks evolve. We leverage the recently published 101 Drosophilidae genome project to analyze the evolution and co-evolution of 11 core clock proteins across 65 species spanning about 60 million years of evolution. A sliding window analysis of coding regions reveals substantial heterogeneity in nucleotide divergence, with *Clk* and *per* exhibiting high divergence, whereas *Pdp1* and *sgg* show virtually no evolutionary change. Additionally, we assessed interdependent amino acid evolution across different proteins, identifying 67 co-evolving site pairs, primarily among CLK-PER, CLK-CWO, and SGG-PER. Using codon-based models of evolution, we found four genes (*cwo, jet, per,* and *sgg*) showing evidence of positive selection. Since several clock proteins are pleiotropic, we tested whether their multifunctionality influences their evolutionary constraints. Using alternative approaches to assess pleiotropy, we found no significant correlation between pleiotropy and the nonsynonymous substitution rate (Ka) in 440 *Drosophila* proteins, including circadian clock ones. Overall, our findings suggest that the circadian clock network does not impose strong constraints on the evolution of its components. This flexibility may facilitate species-specific adaptation of the clock and allow the pleiotropic functions of clock proteins.

Significance Gene networks underlie complex traits and are often thought to constrain molecular evolution because of their interconnected structure and the pleiotropic components. Yet, how such constraints shape the evolution of entire gene networks remains largely unexplored. Here, we present the first comprehensive evolutionary analysis of the full circadian clock protein network across 65 *Drosophila* species. By examining sequence divergence, molecular co-evolution, and pleiotropy across all core clock proteins, we uncover substantial variation in evolutionary rates and identify co-evolving sites between proteins. Strikingly, we show that even highly pleiotropic clock proteins do not exhibit reduced sequence evolution, challenging the assumption that multifunctionality imposes a strong evolutionary constraint. Our findings reveal that the circadian clock network permits a surprising degree of evolutionary flexibility, offering new insights into how conserved biological systems evolve at the molecular level.

## Introduction

In multicellular organisms such as animals and plants, the number of phenotypic states far exceeds the number of proteins within their proteomes. This expansion of phenotypic diversity has been achieved through the evolution of complex gene and protein networks (Greenspan 2009). Mechanisms such as gene duplications enable proteins to acquire novel functions, thereby increasing the diversity of interactions within biological systems (Mottes et al. 2021). Gene duplications not only expand the protein network but also promote the development of new phenotypes, as duplicated genes diverge in function over time. Furthermore, mutations in existing proteins, referred to as link dynamics, serve as a dominant evolutionary force shaping network structure (Berg et al. 2004). The interplay between gene duplications, link dynamics, and stochastic variations in gene expression contributes to the rich diversity of protein networks. These processes illustrate how gene networks evolve to generate new phenotypes and enhance biological diversity through structural and functional innovations.

The circadian clock (Latin for circa “around” and diem “day”) is an endogenous pacemaker that drives diel physiological and biochemical rhythms. The circadian system allows organisms to track the predictable daily changes in their environment, such as light and temperature, resulting from Earth's self-rotation. This ability to anticipate environmental changes and synchronize behavior with optimal times of day provides a strong adaptive advantage. For example, the movement of sunflowers toward the east to face the rising sun and honeybee visits to specific flowers at particular times are both governed by circadian rhythms (Bloch et al. 2017).

Much of the understanding of the molecular circuit underpinning the circadian system has emerged from research on the fruit fly *Drosophila melanogaster*. The first clock gene to be discovered in the fruit fly was *period* (*per*), which, like the following genes that have been discovered, is highly conserved in all other animal groups, including humans.

**Fig. 1.**
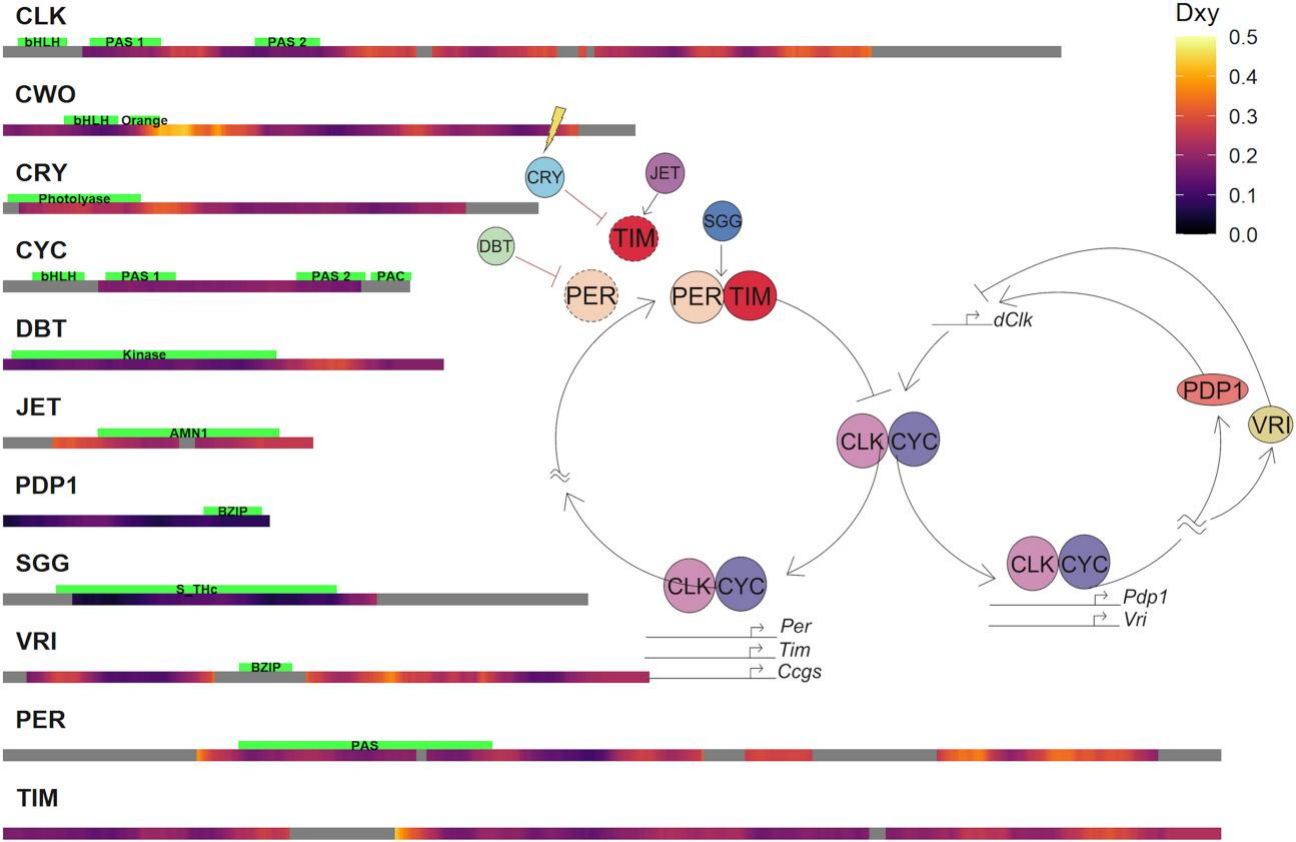
Nucleotide divergence of circadian clock genes across *Drosophila* species. Heatmap showing relative pairwise genetic divergence (Dxy) using a 150 bp sliding window. Grey regions indicate areas of low-quality alignment, where divergence estimates could not be reliably calculated. Inset: Schematic representation of the circadian clock molecular circuit, illustrating the core feedback loops and interactions among clock genes. Arrows indicate activating interactions, whereas blunt-ended lines indicate inhibitory interactions.

The *Drosophila* circadian clock consists of two interlocking negative feedback loops (Fig. 1). In the core loop, CLOCK (CLK) and CYCLE (CYC) form a heterodimer that binds E-box elements to activate transcription of *period* (*per*) and *timeless* (*tim*), whose protein products PER and TIM subsequently form a complex that represses CLK–CYC activity, thereby completing the feedback cycle (Glossop et al. 1999). This repression is reinforced by CLOCK-WORK ORANGE (CWO), which binds E-box regions together with CLK–CYC (Zhou et al. 2016).

The stability and timing of PER and TIM are modulated by phosphorylation through kinases, including DOUBLETIME (DBT) and SHAGGY (SGG). A secondary regulatory loop operates in parallel, in which Vrille (VRI) represses Clk transcription, while PAR-domain protein 1ε (PDP1ε) activates it. Clock output is conveyed to downstream processes via factors such as the pigment-dispersing factor (PDF) neuropeptide and its receptor (PDFR). Entrainment of the circadian clock to the light–dark cycle is mediated by CRYPTOCHROME (CRY), a blue-light photoreceptor, which promotes light-dependent regulation of TIM (Ceriani et al. 1999) through JETLAG (JET), contributing to synchronization with environmental cues.

The circadian clock network is particularly interesting because several clock proteins are pleiotropic, serving roles beyond circadian regulation. For instance, CRY is involved in magnetosensitivity (Gegear et al. 2008), CRY and PDF influence geotaxis (Toma et al. 2002), and SGG participates in the Wnt signaling pathway, essential for development and organogenesis (Papadopoulou et al. 2004). Given their broad functions, pleiotropic genes are often subject to purifying selection, reducing genetic variation (Barbitoff et al. 2025). However, pleiotropy can also lead to balancing selection, maintaining genetic diversity by favoring different alleles for their effects on distinct traits (Mérot et al. 2020).

Previous studies have explored genetic variation in clock genes across different *Drosophila* species, demonstrating how the evolution of these genes drives molecular adaptations specific to the species’ ecological niches (Tauber et al. 2003). These studies have typically focused on the evolution of individual genes within the circadian clock, often in isolation. However, these genes operate as part of a highly interconnected network, and the evolution of the clock system has not been studied as a whole network of interacting proteins. The recent genome sequencing of 93 *Drosophila* species (Kim et al. 2021) allows for a comprehensive analysis of the evolution of circadian clock genes across multiple species. These species represent approximately 60 million years of evolutionary history and span 14 subgroups within *Drosophila* and *Sophophora* (Russo et al. 2013). Here, we analyzed this dataset and identified the orthologous clock proteins across all these species, providing a unique opportunity to examine the evolution of circadian clock genes not in isolation, but as part of a broader, interconnected gene network. One of the key questions addressed in this study is how pleiotropic genes within the circadian clock network evolve, and how their interactions with other genes in the network contribute to the broader evolutionary dynamics of gene networks.

## Results

### Sequence Divergence of Circadian Clock Genes

To assess how individual components of the circadian clock network have evolved, we began by examining patterns of nucleotide divergence across 11 core clock genes in 65 *Drosophila* species. We calculated pairwise nucleotide divergence (Dxy), which measures the average number of nucleotide differences per site between species, using a sliding window approach to capture local variation along each gene. This allowed us to identify both conserved and rapidly evolving regions. The analysis revealed substantial variation between and within genes (Fig. 1). Among the different genes, *cwo* and *tim* exhibited the highest levels of variability, with *tim* displaying the widest range of Dxy values, followed closely by *cwo*. These genes showed substantial fluctuations across their coding regions, indicating a high degree of localized molecular polymorphism. *Clk* and *per* also exhibited relatively high levels of diversity, with mean Dxy values around 0.23 and broad distributions spanning 0.105 to 0.379 and 0.137 to 0.344, respectively.

In contrast, *Pdp1* and *sgg* were the most conserved genes in terms of nucleotide divergence. *Pdp1* had the lowest mean Dxy (0.10) with a narrow distribution ranging from 0.051 to 0.155. Similarly, *sgg* exhibited a low mean Dxy (0.10) and the smallest minimum value (0.04), indicative of strong sequence conservation. *cyc* and *dbt* also demonstrated lower diversity, with mean divergence values around 0.17 and relatively stable distributions.

Variation within genes showed different patterns, with some genes, such as *jet* and *cry,* displaying relatively uniform levels of polymorphism across their length, whereas others, particularly *cwo* and *tim*, showed distinct regions of elevated diversity interspersed with conserved stretches. *Clk*, *per*, and *vri* exhibited intermediate levels of variation, with notable peaks and troughs in diversity along their coding sequences.

### Coevolution of Amino Acids in Clock Proteins

**Fig. 2.**
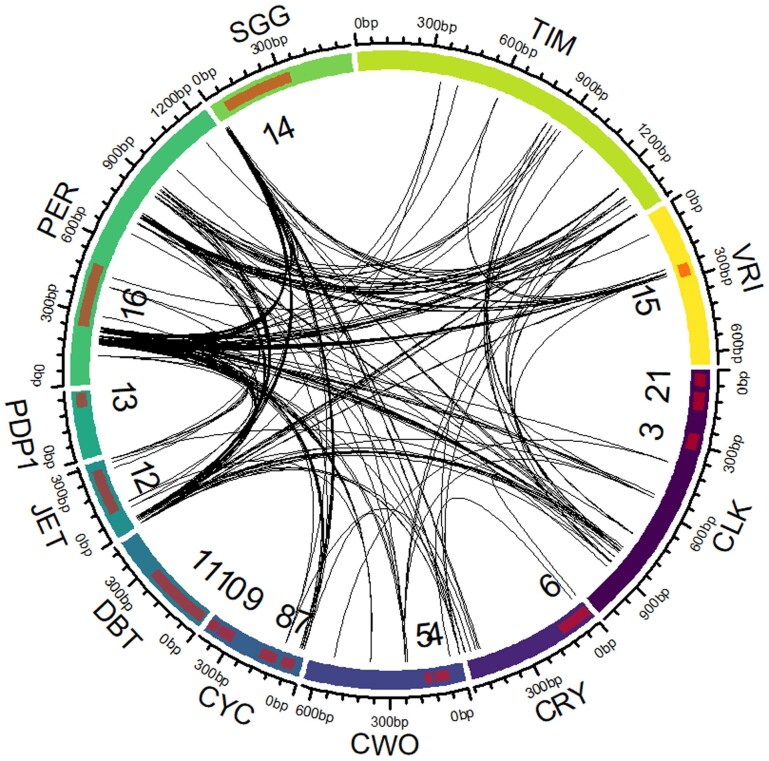
Co-evolving sites among circadian clock proteins. The circular plot shows significant co-evolving pairs of amino acids (p < 0.05). Highlighted sections represent functional domains: 1. bHLH; 2. PAS1; 3. PAS2; 4. bHLH; 5. Orange; 6. Photolyase; 7. bHLH; 8. PAS1; 9. PAS2; 10. PAC; 11. Kinase; 12. AMN1; 13. BZIP; 14. S_THc; 15. BZIP; 16. PAS.

To explore potential functional dependencies between clock proteins, we analyzed coevolution at the amino acid level using CAPS2 (Fares and McNally 2006). This method identifies pairs of residues across proteins that exhibit correlated evolutionary changes across species, which may indicate direct physical interactions, compensatory substitutions, or shared selective pressures. The analysis identified 304 significant co-evolving sites at FDR < 0.05 (Fig. 2, Table S1), spanning 29 unique protein combinations. The strongest signal involved PER, which formed dense networks with several partners. PER-JET accounted for 59 site pairs, followed by PER-TIM (41 pairs), PER-SGG (34 pairs), PER-CLK (25 pairs), and PER-VRI (25 pairs). Secondary hubs included JET-SGG (13 pairs), CYC-SGG, CLK-JET, CLK-TIM, and CWO-PER (12 pairs each), plus PER-CYC and JET-TIM (9 pairs each). Six combinations, including CRY-CWO, CRY-JET, CLK-CRY, and CLK-VRI, were represented by a single co-evolving site.

The distribution of co-evolving sites was not uniform across the sequences, with some regions exhibiting a higher density of co-evolutionary links. Notably, no co-evolving sites were identified in protein pairs that are known to form heterodimers, such as CRY and TIM (Ceriani et al. 1999) or CLK and CYC (Liu et al. 2017). The lack of apparent co-evolution between physically interacting proteins or domains is consistent with recent work showing that compensatory coevolution contributes weakly to evolutionary rate covariation compared with shared selective pressures (Little et al. 2024).

As expected, the number of intra-protein coevolving sites is substantially higher than the number of inter-protein sites (Fig. S1). PER and TIM have the highest number of intra-protein site pairs (506 and 444, respectively), and PDP1 and DBT have the smallest number of intra-protein interactions (6 and 30).

### Selection Analysis

**Fig. 3.**
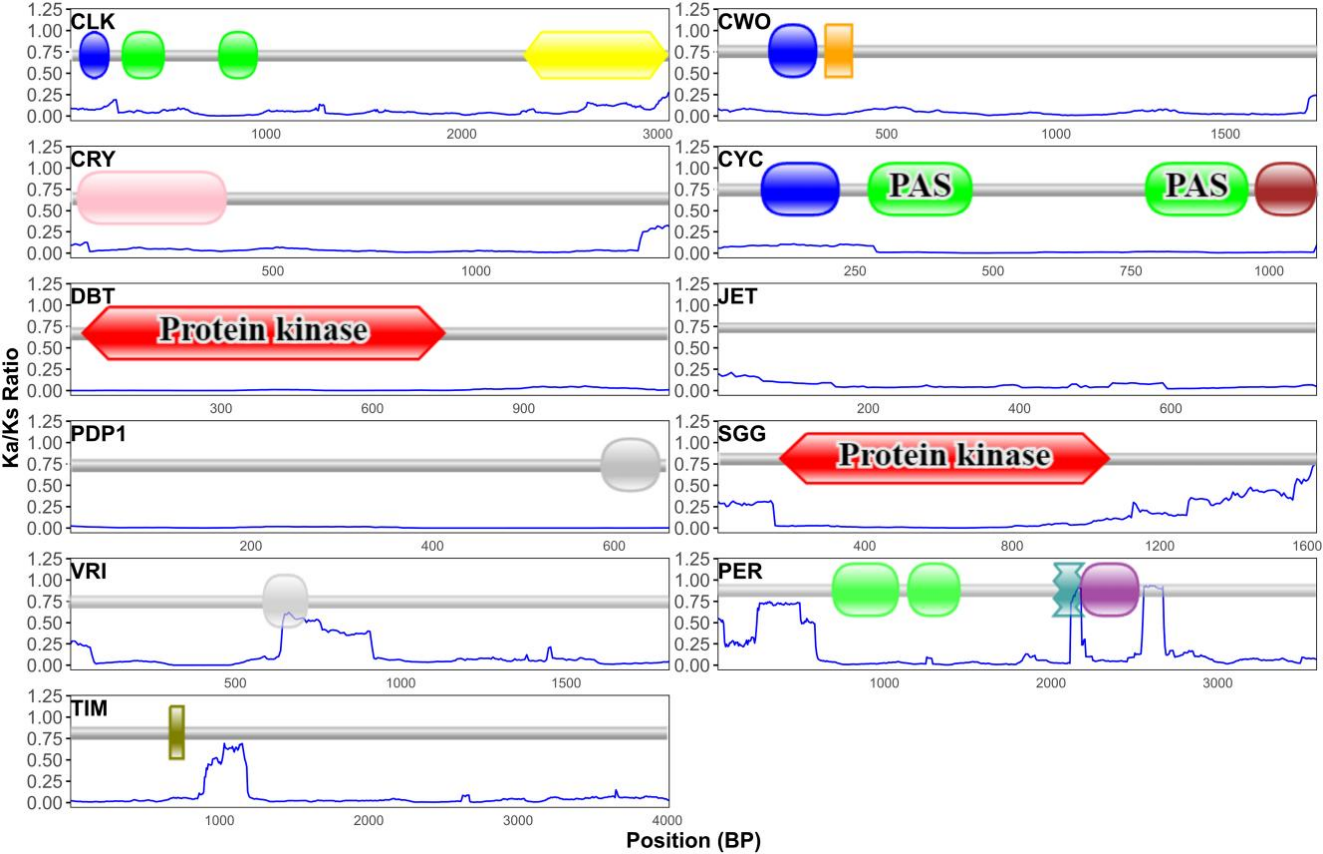
Comparative analysis of selection pressures across *Drosophila* circadian clock genes using sliding window analysis of Ka/Ks ratios. The ratio of non-synonymous to synonymous substitution rates (ω) was calculated to identify signatures of selection.

To investigate patterns of selection pressure across the 11 circadian clock genes, we conducted a sliding window analysis to estimate the Ka/Ks ratio (*ω*) along the length of each gene. The Ka/Ks ratio compares the rate of nonsynonymous (amino acid–changing) substitutions to synonymous (silent) substitutions, providing insight into whether a gene is evolving under purifying, neutral, or positive selection. This analysis revealed considerable variation in selective pressure along the coding regions of different genes (Fig. 3). The majority of genes exhibited consistently low Ka/Ks ratios (*ω* << 1) throughout their lengths, indicative of strong purifying selection. These genes included *Clk*, *cry*, *cyc*, *dbt*, *pdp1*, *tim*, and *vri*. This suggests a strong evolutionary constraint, indicating high conservation and a small number of changes. While no genes displayed Ka/Ks ratios exceeding unity, suggesting pervasive positive selection, some genes exhibited regions with slightly elevated Ka/Ks ratios compared to the baseline. For *per*, the sliding window analysis showed peaks in ω within certain regions along the length of the sequence. A trend toward an increasing ω was observed toward the 3′ end in *sgg*, potentially suggesting relaxed evolutionary selection in this part of the gene. Subtle peaks in Ka/Ks ratios were also apparent along the lengths of *cwo* and *jet*, hinting at localized selective pressures.

To rigorously test for evidence of site-specific selection, we employed codon-based maximum likelihood models implemented in the PAML package (Yang 2007). We note that although these models operate at the codon level, they ultimately infer selection on the encoded amino acids, which determine protein structure, function, and potential interaction sites. Specifically, we compared two nested models: M7, which assumes that sites evolve under a beta distribution of *ω* values strictly less than 1 (i.e. purifying or neutral selection), and M8, which includes an additional category of sites with *ω* > 1, thus allowing for positive selection. A significant result from the likelihood ratio test (LRT) comparing M7 and M8 indicates that allowing for a class of positively selected codons provides a better fit to the data. The results of these analyses are summarized in Table 1.

**Table 1:** Likelihood ratio tests (LRTs) for positive selection in *Drosophila* circadian clock genes

Gene	Model	df	LRT (2Δℓ)	*P*-value
CLK	M0 to M1	1	713.49	0.0
	M7 to M8	2	−0.02	1.0
CRY	M0 to M1	1	501.19	0.0
	M7 to M8	2	−0.01	1.0
CWO	M0 to M1	1	628.18	0.0
	M7 to M8	2	7.07	0.029*
CYC	M0 to M1	1	45.74	0.0
	M7 to M8	2	−0.01	1.0
DBT	M0 to M1	1	99.87	0.0
	M7 to M8	2	−0.01	1.0
JET	M0 to M1	1	491.97	0.0
	M7 to M8	2	9.39	0.009*
PDP1	M0 to M1	1	7.51	0.006
	M7 to M8	2	4.39	0.112
PER	M0 to M1	1	2008.64	0.0
	M7 to M8	2	6.07	0.048*
SGG	M0 to M1	1	2282.52	0.0
	M7 to M8	2	22.94	0.0***
TIM	M0 to M1	1	1802.13	0.0
	M7 to M8	2	1.13	0.567
VRI	M0 to M1	1	717.37	0.0
	M7 to M8	2	−0.0	1.0

The table summarizes the results of PAML analyses comparing different codon substitution models to detect positive selection. The model comparisons shown are M0vsM1 (one ratio vs. multiple ratio), and M7 vs M8 (beta vs. beta and positive).

The LRT comparing models M7 and M8 revealed statistically significant evidence of positive selection in 4 of the 11 genes examined: *cwo*, *jet*, *per*, and *sgg* (*P* < 0.05). This finding suggests that these genes have experienced adaptive evolution at specific codon sites. No significant evidence of positive selection was detected in *Clk*, *cry*, *cyc*, *dbt*, *pdp1*, *tim*, or *vri*, which is consistent with the strong purifying selection observed in the sliding window analysis. The genes with significantly higher LRT results are *cwo*, *jet*, *per*, and *sgg*. This supports the sliding windows result, further indicating these regions have undergone positive selection. The PAML analysis also revealed significant site-specific positive selection in three circadian clock genes: *cwo* (G203)*, per* (G909), and *sgg* (G442; S452). The latter site is particularly important since it serves as an additional serine phosphorylation site of SGG in *D. melanogaster*, which is known to be important for circadian function (Beck et al. 2018).

We also tested for site-specific episodic diversifying selection using MEME (Mixed Effects Model of Evolution) (Murrell et al. 2012). MEME allows the nonsynonymous/synonymous rate ratio (*ω*) to vary across branches at each codon, thereby detecting positive selection that affects only a subset of lineages. At a significance threshold of *P* < 0.05, MEME identified numerous codons under episodic selection in most circadian clock genes. The largest numbers of significant sites were found in *sgg* (49 sites), *tim* (38), *per* (36), *Clk* (42), and *vri* (30). Additional genes also showed evidence of episodic selection, including *cwo* (29), *jet* (6), *dbt* (13), and *cry* (11). A smaller number of sites were detected in *Pdp1* (4). Thus, nearly all core components of the circadian clock show signatures of lineage-specific positive selection, with particularly strong episodic signals in *Clk*, *per*, *tim*, *sgg*, and *vri* (Table S2)

### Pleiotropy and Protein Evolution

**Fig. 4:**
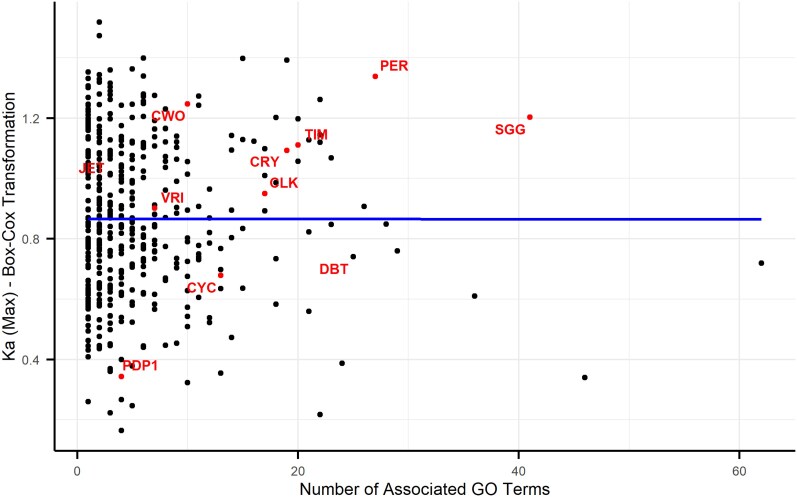
Relationship between evolutionary rate and pleiotropy in *Drosophila* proteins. The nonsynonymous substitution rate (Ka*) was calculated across 440 proteins from 65 *Drosophila* species and plotted against each protein's pleiotropy score, measured as the number of associated GO terms. Each point represents an individual protein, with circadian clock proteins (n=11) highlighted in red. The blue line shows a linear regression fit to the data, which revealed no significant correlation between Ka and pleiotropy score (see text). Note that the x-axis contains a break to optimize the display of the data distribution.

Since some of the clock proteins are known to be pleiotropic, we have sought to explore the link between pleiotropy and protein divergence. To that end, we have analyzed 429 additional, randomly selected proteins from the same 65 *Drosophila* species that were used for the clock protein analysis. The annotation and alignment of these proteins were carried out in the same way as was done with the clock proteins. Using the number of GO terms associated as a measure of pleiotropy underscores this property among clock proteins (Fig. 4). Six clock proteins were amongst the top 10% of the number of associated GO terms, with PER and SGG being the most pleiotropic. Although these two proteins exhibited elevated nonsynonymous substitution rate (Ka), we found no significant link between Ka* and pleiotropy in clock proteins, or generally among the large sample of proteins (*P* = 0.99, Fig. 4). The gene-wise Ka also did not show a significant association with the pleiotropic score (Fig. S2). We have also estimated pleiotropy by the number of interactions that a gene/protein is known to have. Here, the linear model was also not significant (*F*_1,388_ = 1.40, *P* = 0.24), with a low adjusted *R*^2^ (0.001), suggesting again that protein interactions as a pleiotropy measure do not predict the level of Ka* (Fig. S2).

### Incongruency of Protein Phylogenies

**Fig. 5.**
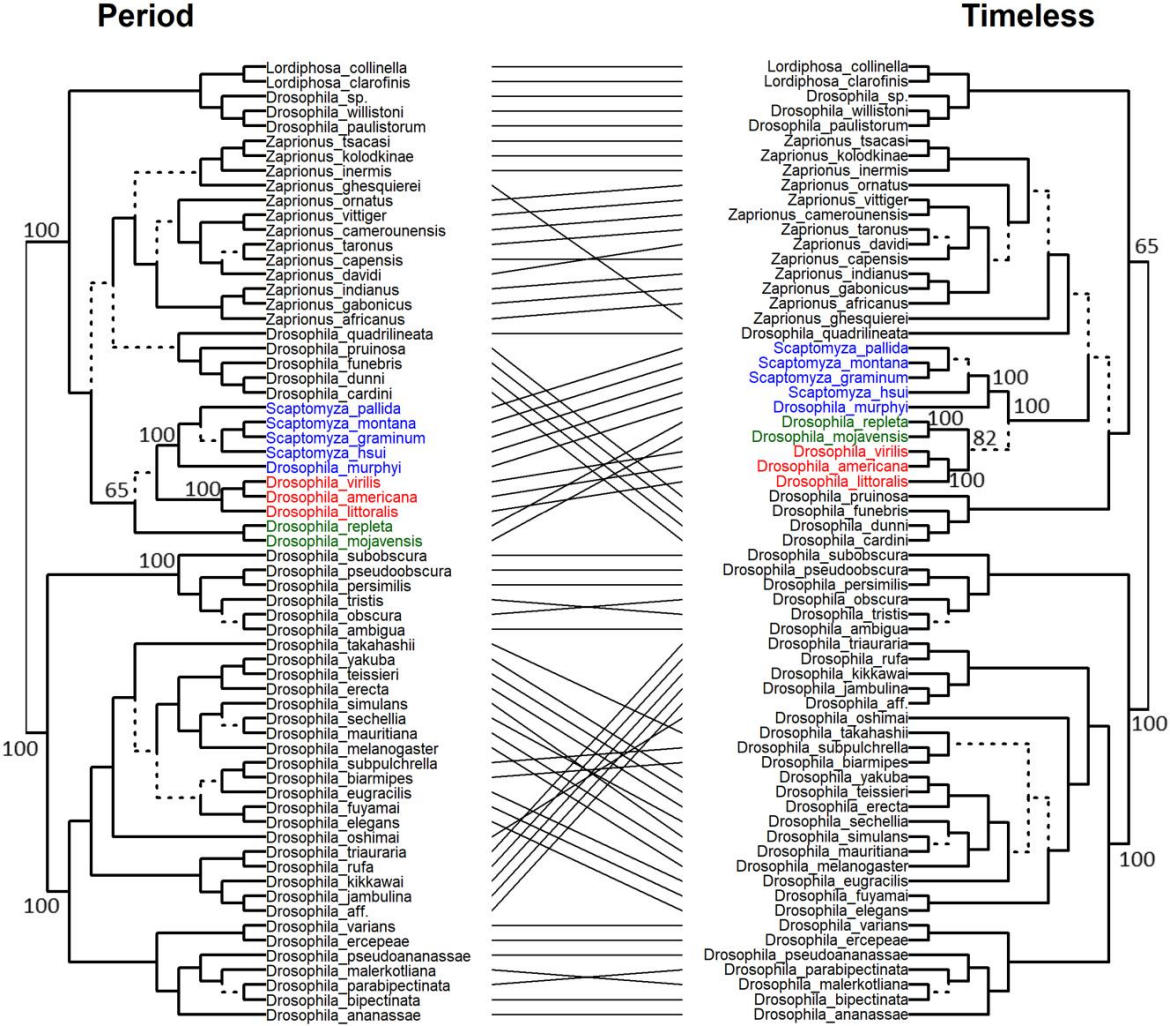
Phylogenetic incongruence between PER and TIM protein trees in *Drosophila*. To examine potential co-evolution between these core circadian clock components, we compared phylogenetic trees inferred from nucleotide alignments of PER and TIM across 65 *Drosophila* species. The mirrored trees (tanglegram) reveal multiple topological incongruences (indicated by crossing lines) suggesting differing evolutionary pressures or rates of change acting on PER versus TIM. Branches leading to distinct subtrees are marked by dashed lines. Bootstrap support values (≥65%) are shown for major internal nodes.

Incongruency between phylogenetic trees of proteins that are members of the same networks can provide valuable insights into the evolution and co-evolution of these proteins. Here, we generated mirror-trees (tanglegrams) of different pairs of clock proteins and assessed the level of incongruency between the dendrograms. The PER-TIM tanglegram shows a substantial incongruency (Fig. 5). For example, in the PER phylogeny, the virilis species group (e.g. *D. virilis*, *D. americana*, *D. littoralis*, shown in red) does not cluster with the repleta group (e.g. *D. repleta*, *D. mojavensis*, shown in green), but instead appears closer to the *Scaptomyza* clade (blue), thereby disrupting the monophyly of the subgenus *Drosophila*. In contrast, in the TIM phylogeny, the *virilis* and *repleta* groups cluster together, as expected for members of the subgenus *Drosophila*. Thus, PER's phylogeny fails to unite the *virilis* and *repleta* clades, a deviation previously noted in smaller datasets (Noreen et al. 2018).

The PER-CRY tanglegram also revealed several major topological incongruences (Fig. S3). In the CRY tree, species from the *virilis* and *repleta* groups form a single clade, consistent with their classification within subgenus *Drosophila*. The subgenus *Drosophila* comprises several major species groups, including the virilis and repleta groups, whereas *Scaptomyza* represents a more distantly related lineage that falls outside this subgenus. These relationships are well supported by accepted *Drosophila* species trees (Russo et al. 2013), which provide the phylogenetic framework for the colored clades shown in Fig. 5. In contrast, these groups are split in the PER tree, indicating disrupted monophyly. Another major difference involves the *eugracilis–biarmipes* lineage, which forms a clade in the PER tree but is broken in the CRY tree, where *D. eugracilis* is grouped with the *elegans* subgroup instead. These inconsistencies, especially where clades are clearly broken in one tree but intact in the other, suggest that PER and CRY have followed distinct evolutionary paths in some lineages, possibly reflecting different functional constraints or adaptive pressures. Substantial incongruencies are also present in the CLK-CYC, CLK-CWO and CRY-TIM tanglegrams (Fig. S4 to S6).

## Discussion

Here, we measured the evolutionary rates shaping the circadian clock network. Focusing on coding DNA sequences, our implicit hypothesis was that species-specific adaptations to local environmental conditions would be mirrored by the pattern of molecular divergence and substitution rate that various core clock genes undergo.

Previous studies showed that variation in a single clock gene may modify circadian function. For example, diel locomotor and sexual activity profiles differ between *D. melanogaster* and *D. psuedoobscura*, and these differences are solely due to a species variation in the *per* gene as was demonstrated by comparing the behavior of transgenic *D. melanogaster* flies that harbored either the hetero- or the conspecific *per* gene (Tauber et al. 2003). However, in a different study, the rescue of the circadian function of *per* mutant *D. melanogaster* by different species *per* transgene yielded different levels of rescue, and this was explained by PER-TIM co-evolution (Piccin et al. 2000). Later experiments with two heterospecific transgenes expressing PER and TIM together in the double mutant *D. melanogaster* flies provided weak or partial evidence for co-evolution (Noreen et al. 2018). Similarly, the TIM transgene from remotely related species *D. ananassae* was able to rescue the circadian rhythm of D. *melanogaster* mutants (Nishinokubi et al. 2003), alluding to a weak constraint of co-evolution of TIM with other network proteins. These experiments are consistent with our finding here of modest molecular co-evolution between network proteins, which was notably not evident in pairs that are physically interacting, such as PER and TIM, or CLK and CYC (Fig. 2).

We acknowledge that the genetic variation in the core clock genes may capture only a small amount of the variation that drives local adaptation. Variation in other clock-controlled genes may contribute to variation in the circadian phenotypes, such as the phase, as was demonstrated in studies in *D. melanogaster* (Pegoraro et al. 2015, 2020), or in GWAS in human populations (Hu et al. 2016). Furthermore, some molecular adaptations in nonclock genes may modify the neuronal wiring of the clock neurons in the brain during development (rather than the cell-autonomous TTFL circuit). Species-specific wiring variations may lead to different neurochemistry of the circadian network, which may underlie the species local adaptation. For example, a study comparing *D*. *ezoana* and *D*. *littoralis*, from Northern Europe, to *D*. *melanogaster*, an equatorial strain, revealed expression patterns of PDF and CRY in clock and nonclock neurons that correlated with the different circadian behavior of high- and low latitudinal species (Menegazzi et al. 2017).

A central hypothesis in evolutionary biology is that pleiotropy, where a single gene influences multiple traits, constrains sequence evolution. Because mutations in such genes can have widespread deleterious effects, they are expected to be under strong purifying selection and evolve slowly, a pattern observed across diverse taxa (He and Zhang 2006; Hu et al. 2017; Mähler et al. 2017; Martin and Tate 2023).

Contrary to this expectation, our analysis revealed no significant correlation between a gene's pleiotropic degree and its rate of protein evolution (Ka). Highly pleiotropic genes like *sgg* or *Pdp1* did not evolve more slowly than their less pleiotropic counterparts. We acknowledge, however, that both of our pleiotropy metrics (GO term annotation and gene interaction counts) are imperfect proxies. GO terms can be biased by differential annotation effort, and interaction databases may underrepresent certain pathways. Therefore, our conclusion refers to a lack of association between Ka and these proxies for pleiotropy, rather than pleiotropy itself.

This lack of association suggests that the impact of pleiotropy on the clock network is more nuanced than often assumed. One explanation is modular pleiotropy, where distinct protein domains compartmentalize different functions. This architecture may permit adaptive evolution in specific regions, such as those tuning circadian rhythms, without disrupting other vital roles, thus relaxing the overall sequence constraint (Chesmore et al. 2016).

Our findings highlight that the circadian clock network, despite its interwoven architecture and pleiotropic components, does not enforce an unusually slow evolutionary rate on its genes. This contrasts with a simplistic view that pleiotropy invariably slows evolution, and instead hints at an inherent flexibility in how clock genes balance multiple roles. One interpretation is that adaptive evolution in clock genes might occur through mechanisms other than protein-coding change, such as cis-regulatory modifications or gene expression adjustments, thereby sidestepping the pleiotropic constraints on the protein sequence. Zhang (2023) noted that evolutionary adaptation can sometimes be channeled through regulatory changes in pleiotropic genes to avoid detrimental effects on other traits. In the circadian system, species-specific adaptations of rhythms could predominantly involve changes in gene regulation or network interactions, allowing core clock protein sequences to drift more freely than expected. In the circadian clock, species-specific enhancer changes in *pigment-dispersing factor* (*Pdf*) abolish photoperiodic plasticity in *D. sechellia*, and the same variants shift evening activity in *D. melanogaster* (Shahandeh et al. 2024). Population genomic analyses reveal signals of directional selection on *Pdf* enhancers in *D. sechellia* and latitudinally varying selection in high-latitude *D. melanogaster* populations, illustrating how adaptive noncoding changes can reshape behavior without altering the protein.

Another source of variation we did not examine is alternative splicing. In *D. melanogaster,* the clock gene *per* yields two transcripts; warm temperatures favor the intron-retaining form, shortening the circadian period, whereas cool temperatures promote intron removal and lengthen it (Majercak et al. 1999). This thermal switch is weak in *D. yakuba*, showing that small regulatory changes rather than coding differences can adjust timing across species (Low et al. 2008). A similar “molecular thermometer” exists in *timeless*: cold favors *tim-cold* and *tim-short-and-cold*, heat elevates *tim-medium*, and toggling among them fine-tunes TIM levels (Anduaga et al. 2019). Close relatives retain this response, whereas more distant flies modify or lose it, and lineage-specific gains or losses of the responsive exons are seen across Diptera (Bullo et al. 2025). Thus, alternative splicing offers a versatile, noncoding route for thermal adaptation of circadian behavior. Because our study relied solely on genome sequences, we could not assess splice isoforms; resolving their evolutionary dynamics will require a comprehensive cross-species transcriptomic resource comparable in scope to current genomic datasets.

In addition to rate-based analyses, our tanglegram comparisons revealed notable phylogenetic incongruence between several clock-interacting proteins, including PER and TIM, as well as PER and CRY. While such discordance may result from incomplete lineage sorting, introgression, or differential selection pressures, the lack of detailed biological and ecological data, particularly regarding circadian behavior and photoperiodic adaptation for most *Drosophila* species, limits our ability to interpret these patterns confidently. Phylogenetic incongruence among gene trees is not uncommon, but in this case, further comparative work linking the evolution of clock proteins to species-specific traits would be needed to assess possible selective mechanisms.

In summary, the circadian clock genes provided an empirical test of the long-held assumption that pleiotropy constrains molecular evolution. Contrary to expectations from earlier studies and theoretical models, we found no evidence that pleiotropic clock genes evolve more slowly at the sequence level. This divergence from prior patterns underscores the complexity of evolutionary dynamics in protein networks. It suggests that pleiotropy's impact on sequence divergence is context-dependent and may be mitigated by modular functionality or alternative adaptive pathways. Our results contribute to a growing recognition that gene network evolution can accommodate pleiotropic versatility without universally imposing strong evolutionary constraint, thereby allowing even multifunctional proteins like clock components to explore sequence space and potentially facilitate lineage-specific innovations in circadian biology.

## Materials and Methods

### Gene Prediction and Protein Alignment

The 101 Drosophila genome project consisted of 101 genome scaffolds (Kim et al. 2021), lacking gene annotation. To identify the Drosophila 11 clock proteins (Fig. 1), we used AUGUSTUS software for gene prediction (Stanke et al. 2006). The search for the orthologous gene was facilitated using *D. melanogaster* sequences. These sequences were used in a BLAST search against a database of every genome from the 101 Drosophila genomes project. These BLAST fragments were then used to search for the predicted genes from the respective species. The collected sequences were aligned using MAFFT v7.490 (Katoh et al. 2002). These alignments are then scored using Alistat (Wong et al. 2020), where species sequences that had less than 40% consensus were removed. The sequences were converted to amino acid sequences and were re-aligned using the codon information. Finally, the alignments were adjusted by visual inspection. The final alignment included sequences from 65 different *Drosophila* species.

### DNA Variation Analysis

To assess interspecific genetic variation, we calculated the average pairwise nucleotide divergence across *Drosophila* species, analogous to the *π* statistic. To that end, we used the *pegas* R package (Paradis and Barrett 2010). To minimize the sites with gaps in the data, we carried out pruning of the alignments, where sequences with a high percentage of gaps (over 5% of the total sequence length, or over 100 gap bases total) were removed. Using a custom code, the polymorphism was computed for a sliding window of a 180 bp window length, and a 6 bp step size. The output of the analysis was exported as text files and heatmap plots depicting polymorphism along the coding regions were created.

We carried out a nonsynonymous-to-synonymous substitution ratio test (the *K*_A_/*K*_S_ test, *ω*) using a custom-made script and the *SeqinR* R package (Charif and Lobry 2007). The script runs a sliding window of 150 bp and a 6 bp step. This analysis provides a visual representation of the variation in selection pressure, with *ω* < 1 indicating purifying selection, *ω* ≈ 1 indicating neutral evolution, and *ω* > 1 suggesting positive selection. Graphs were made using the R software.

To test for the signature of negative purifying selection or accelerated evolution driven by positive Darwinian selection, we used the CodeML program from the PAML package (Yang 2007). We used the models M0 (One-ratio model), M1 (Nearly neutral model), M2 (Positive selection model), M7 (Beta model), and M8 (Beta & *ω* > 1 model). We compared the models using a likelihood ratio test. A custom script was made to conduct the model comparison and parse the significant sites from the results files.

We tested for site-wise episodic diversifying selection using MEME (Mixed Effects Model of Evolution) as implemented in HyPhy (Kosakovsky Pond et al. 2020). MEME fits a mixed-effects codon model that allows the nonsynonymous/synonymous rate ratio (*ω*) to vary across branches at each site, enabling detection of positive selection acting on a subset of lineages (episodic selection). We used the same codon alignments and gene trees as for the PAML analyses, ran MEME with default settings, and considered sites significant at *P* ≤ 0.05 (likelihood ratio test) unless otherwise specified.

We used the AutoCoEv pipeline (Petrov et al. 2022), which employs a modified version of the CAPS2 software (Fares and McNally 2006) to predict co-evolution between proteins. Notably, the patched version of CAPS2 provides extended statistical output, enabling correction for multiple comparisons. As orthologs had already been identified, we skipped the initial steps of AutoCoEv and proceeded directly from the prepared multiple sequence alignments (MSAs). Phylogenetic trees were generated using PhyML v3.3.20250515 (Guindon et al. 2010) and parsed into CAPS2, which was run with threshold settings of α = 0.05 and bootstrap = 0.95. We considered sites with Benjamini–Hochberg adjusted *P*-values (FDR) below 0.05 as statistically significant. The R package *circlize* was used to create the circular graphs (Gu et al. 2014).

To assess the pleiotropy of *Drosophila* genes, we used two distinct methods. The first utilized the DRoID interactome database (Murali et al. 2011). A custom-written code screened the database files and counted the number of times a gene of interest interacted with other genes. The database files that were used included yeast-2-hybrid files and the Flybase genetic interaction database (duplicate interactions were counted once).

The second method of quantifying pleiotropy was based on a gene of interest's number of Gene Ontology (GO) terms. Only the highest-level terms of the GO hierarchy (“Biological Process”) were counted. The consistency of these methods was compared using R. The rate of non-silent substitutions (*K*_a_) across species was calculated as described above, and the effect of pleiotropy was tested using (i) the gene-wide overall *K*_a_ (ii) the maximum *K*_a_ (*K*_a_*) of a window with few gaps (<1,000 of 6,630) from the sliding window analysis. A linear model was carried out in R using the MASS library with a Box-Cox transformation to stabilize the variance of the Ka data.

### Phylogenetic Trees and Tanglegrams

Gene trees were generated with RAxML using default settings (Stamatakis 2014). These were imported into R. Using the ape R library (Paradis and Schliep 2019), the tree was converted to an ultrametric tree using the *chronos()* function. The “mirror-trees” (tanglegrams) of pairs of clock proteins were generated using the *dendextend* library (Galili 2015), and their co-evolution was visually analyzed.

## Supplementary Material

evag061_Supplementary_Data

## Data Availability

All data supporting this study have been deposited in Zenodo (https://doi.org/10.5281/zenodo.15187785). This repository includes predicted gene files, multiple sequence alignments, and the complete input and output files from AUGUSTUS, CAPS, CodeML, and the pleiotropy analysis. The computational pipeline scripts used for analysis are available in our GitHub repository (https://tinyurl.com/25xmsbkt).

## References

[evag061-B1] Anduaga AM, et al Thermosensitive alternative splicing senses and mediates temperature adaptation in *Drosophila*. Elife. 2019:8:e44642. 10.7554/ELIFE.44642. 31702556 PMC6890466

[evag061-B2] Barbitoff YA, Bogaichuk PM, Pavlova NS, Malysheva PV, Predeus AV. Functional determinants and evolutionary consequences of pleiotropy in complex and Mendelian traits. Mol Biol Evol. 2025:42:msaf232. 10.1093/molbev/msaf232. 40974070 PMC12534788

[evag061-B3] Beck K, Hovhanyan A, Menegazzi P, Helfrich-Förster C, Raabe T. *Drosophila* RSK influences the pace of the circadian clock by negative regulation of protein kinase *shaggy* activity. Front Mol Neurosci. 2018:11:361205. 10.3389/FNMOL.2018.00122/BIBTEX. PMC5908959 29706866

[evag061-B4] Berg J, Lässig M, Wagner A. Structure and evolution of protein interaction networks: a statistical model for link dynamics and gene duplications. BMC Evol Biol. 2004:4:1–12. 10.1186/1471-2148-4-51/FIGURES/4. 15566577 PMC544576

[evag061-B5] Bloch G, Bar-Shai N, Cytter Y, Green R. Time is honey: circadian clocks of bees and flowers and how their interactions may influence ecological communities. Philos Trans R Soc B Biol Sci. 2017:372:20160256. 10.1098/RSTB.2016.0256. PMC5647282 28993499

[evag061-B6] Bullo E, Chen P, Fiala I, Smýkal V, Doležel D. Coevolution of *Drosophila*-type *timeless* with partner clock proteins. iScience. 2025:28:112338. 10.1016/J.ISCI.2025.112338. 40322083 PMC12049834

[evag061-B7] Ceriani MF, et al Light-dependent sequestration of TIMELESS by CRYPTOCHROME. Science. 1999:285:553–556. 10.1126/science.285.5427.553. 10417378

[evag061-B8] Charif D, Lobry JR. Seqinr 1.0-2: a contributed package to the R project for statistical computing devoted to biological sequences retrieval and analysis. In: Bastolla U, Porto M, Roman HE, Vendruscolo M, editors. Structural approaches to sequence evolution. Biological and medical physics, biomedical engineering. Springer; 2007. p. 207–232. 10.1007/978-3-540-35306-5_10.

[evag061-B9] Chesmore KN, Bartlett J, Cheng C, Williams SM. Complex patterns of association between pleiotropy and transcription factor evolution. Genome Biol Evol. 2016:8:3159–3170. 10.1093/GBE/EVW228. 27635052 PMC5174740

[evag061-B10] Fares MA, McNally D. CAPS: coevolution analysis using protein sequences. Bioinformatics. 2006:22:2821–2822. 10.1093/BIOINFORMATICS/BTL493. 17005535

[evag061-B11] Galili T . Dendextend: an R package for visualizing, adjusting and comparing trees of hierarchical clustering. Bioinformatics. 2015:31:3718. 10.1093/BIOINFORMATICS/BTV428. 26209431 PMC4817050

[evag061-B12] Gegear RJ, Casselman A, Waddell S, Reppert SM. Cryptochrome mediates light-dependent magnetosensitivity in Drosophila. Nature. 2008:454:1014–1018. 10.1038/NATURE07183;KWRD=SCIENCE. 18641630 PMC2559964

[evag061-B13] Glossop NRJ, Lyons LC, Hardin PE. Interlocked feedback loops within the *Drosophila* circadian oscillator. Science. 1999:286:766–768. 10.1126/science.286.5440.766. 10531060

[evag061-B14] Greenspan RJ . Selection, gene interaction, and flexible gene networks. Cold Spring Harb Symp Quant Biol. 2009:74:131–138. 10.1101/sqb.2009.74.029. 19903749

[evag061-B15] Gu Z, Gu L, Eils R, Schlesner M, Brors B. Circlize implements and enhances circular visualization in R. Bioinformatics. 2014:30:2811–2812. 10.1093/BIOINFORMATICS/BTU393. 24930139

[evag061-B16] Guindon S, et al New algorithms and methods to estimate maximum-likelihood phylogenies: assessing the performance of PhyML 3.0. Syst Biol. 2010:59:307–321. 10.1093/SYSBIO/SYQ010. 20525638

[evag061-B17] He X, Zhang J. Toward a molecular understanding of pleiotropy. Genetics. 2006:173:1885–1891. 10.1534/GENETICS.106.060269. 16702416 PMC1569710

[evag061-B18] Hu H, et al Constrained vertebrate evolution by pleiotropic genes. Nat Ecol Evol. 2017:1:1722–1730. 10.1038/s41559-017-0318-0. 28963548

[evag061-B19] Hu Y, et al GWAS of 89,283 individuals identifies genetic variants associated with self-reporting of being a morning person. Nat Commun. 2016:7:10448. 10.1038/ncomms10448. 26835600 PMC4740817

[evag061-B20] Katoh K, Misawa K, Kuma KI, Miyata T. MAFFT: a novel method for rapid multiple sequence alignment based on fast Fourier transform. Nucleic Acids Res. 2002:30:3059–3066. 10.1093/NAR/GKF436. 12136088 PMC135756

[evag061-B21] Kim BY, et al Highly contiguous assemblies of 101 drosophilid genomes. Elife. 2021:10:e66405. 10.7554/ELIFE.66405. 34279216 PMC8337076

[evag061-B22] Kosakovsky Pond SL, et al Hyphy 2.5—a customizable platform for evolutionary hypothesis testing using phylogenies. Mol Biol Evol. 2020:37:295–299. 10.1093/MOLBEV/MSZ197. 31504749 PMC8204705

[evag061-B23] Little J, Chikina M, Clark NL. Evolutionary rate covariation is a reliable predictor of co-functional interactions but not necessarily physical interactions. Elife. 2024:12:RP93333. 10.7554/ELIFE.93333. 38415754 PMC10942632

[evag061-B24] Liu T, Mahesh G, Yu W, Hardin PE. CLOCK stabilizes CYCLE to initiate clock function in *Drosophila*. Proc Natl Acad Sci U S A. 2017:114:10972–10977. 10.1073/pnas.170714311. 28973907 PMC5642697

[evag061-B25] Low KH, Lim C, Ko HW, Edery I. Natural variation in the splice site strength of a clock gene and species-specific thermal adaptation. Neuron. 2008:60:1054–1067. 10.1016/J.NEURON.2008.10.048. 19109911 PMC2631419

[evag061-B26] Mähler N, et al Gene co-expression network connectivity is an important determinant of selective constraint. PLoS Genet. 2017:13:e1006402. 10.1371/JOURNAL.PGEN.1006402. 28406900 PMC5407845

[evag061-B27] Majercak J, Sidote D, Hardin PE, Edery I. How a circadian clock adapts to seasonal decreases in temperature and day length. Neuron. 1999:24:219–230. 10.1016/S0896-6273(00)80834-X. 10677039

[evag061-B28] Martin RA, Tate AT. Pleiotropy promotes the evolution of inducible immune responses in a model of host-pathogen coevolution. PLoS Comput Biol. 2023:19:e1010445. 10.1371/JOURNAL.PCBI.1010445. 37022993 PMC10079112

[evag061-B29] Menegazzi P, et al Adaptation of circadian neuronal network to photoperiod in high-latitude European Drosophilids. Curr Biol. 2017:27:833–839. 10.1016/J.CUB.2017.01.036. 28262491

[evag061-B30] Mérot C, Llaurens V, Normandeau E, Bernatchez L, Wellenreuther M. Balancing selection via life-history trade-offs maintains an inversion polymorphism in a seaweed fly. Nat Commun. 2020:11:1–11. 10.1038/s41467-020-14479-7. 32015341 PMC6997199

[evag061-B31] Mottes F, Villa C, Osella M, Caselle M. The impact of whole genome duplications on the human gene regulatory networks. PLoS Comput Biol. 2021:17:e1009638. 10.1371/JOURNAL.PCBI.1009638. 34871317 PMC8675932

[evag061-B32] Murali T, et al DroID 2011: a comprehensive, integrated resource for protein, transcription factor, RNA and gene interactions for *Drosophila*. Nucleic Acids Res. 2011:39:D736–D743. 10.1093/NAR/GKQ1092. 21036869 PMC3013689

[evag061-B33] Murrell B, et al Detecting individual sites subject to episodic diversifying selection. PLoS Genet. 2012:8:e1002764. 10.1371/JOURNAL.PGEN.1002764. 22807683 PMC3395634

[evag061-B34] Nishinokubi I, et al Highly conserved *Drosophila ananassae timeless* gene functions as a clock component in *Drosophila melanogaster*. Gene. 2003:307:183–190. 10.1016/S0378-1119(03)00468-2. 12706901

[evag061-B35] Noreen S, Pegoraro M, Nouroz F, Tauber E, Kyriacou CP. Interspecific studies of circadian genes *period* and *timeless* in *Drosophila*. Gene. 2018:648:106–114. 10.1016/j.gene.2018.01.020. 29353056 PMC5818170

[evag061-B36] Papadopoulou D, Bianchi MW, Bourouis M. Functional studies of shaggy/glycogen synthase kinase 3 phosphorylation sites in *Drosophila melanogaster*. Mol Cell Biol. 2004:24:4909–4919. 10.1128/MCB.24.11.4909-4919.2004. 15143183 PMC416399

[evag061-B37] Paradis E, Barrett J. pegas: an R package for population genetics with an integrated–modular approach. Bioinformatics. 2010:26:419–420. 10.1093/BIOINFORMATICS/BTP696. 20080509

[evag061-B38] Paradis E, Schliep K. ape 5.0: an environment for modern phylogenetics and evolutionary analyses in R. Bioinformatics. 2019:35:526–528. 10.1093/BIOINFORMATICS/BTY633. 30016406

[evag061-B39] Pegoraro M, et al Gene expression associated with early and late chronotypes in *Drosophila melanogaster*. Front Neurol. 2015:6:100. 10.3389/fneur.2015.00100. 26097463 PMC4457141

[evag061-B40] Pegoraro M, et al The genetic basis of diurnal preference in *Drosophila melanogaster*. BMC Genomics. 2020:21:596. 10.1186/s12864-020-07020-z. 32862827 PMC7457780

[evag061-B41] Petrov PB, Awoniyi LO, Šuštar V, Balci MÖ, Mattila PK. Autocoev—a high-throughput in silico pipeline for predicting inter-protein coevolution. Int J Mol Sci. 2022:23:3351. 10.3390/IJMS23063351/S1. 35328772 PMC8952222

[evag061-B42] Piccin A, et al The clock gene *period* of the housefly, *Musca domestica*, rescues behavioral rhythmicity in *Drosophila melanogaster*: evidence for intermolecular coevolution? Genetics. 2000:154:747–758. 10.1093/GENETICS/154.2.747. 10655226 PMC1460960

[evag061-B43] Russo CAM, Mello B, Frazão A, Voloch CM. Phylogenetic analysis and a time tree for a large drosophilid data set (Diptera: Drosophilidae). Zool J Linn Soc. 2013:169:765–775. 10.1111/ZOJ.12062.

[evag061-B44] Shahandeh MP, et al Circadian plasticity evolves through regulatory changes in a neuropeptide gene. Nature. 2024:635:951–959. 10.1038/s41586-024-08056-x. 39415010 PMC11602725

[evag061-B45] Stamatakis A . RAxML version 8: a tool for phylogenetic analysis and post-analysis of large phylogenies. Bioinformatics. 2014:30:1312–1313. 10.1093/BIOINFORMATICS/BTU033. 24451623 PMC3998144

[evag061-B46] Stanke M, et al AUGUSTUS: ab initio prediction of alternative transcripts. Nucleic Acids Res. 2006:34:W435–W439. 10.1093/NAR/GKL200. 16845043 PMC1538822

[evag061-B47] Tauber E, Roe H, Costa R, Hennessy JM, Kyriacou CP. Temporal mating isolation driven by a behavioral gene in *Drosophila*. Curr Biol. 2003:13:140–145. 10.1016/S0960-9822(03)00004-6. 12546788

[evag061-B48] Toma DP, White KP, Hirsch J, Greenspan RJ. Identification of genes involved in *Drosophila melanogaster* geotaxis, a complex behavioral trait. Nat Genet. 2002:31:349–353. 10.1038/ng893. 12042820

[evag061-B49] Wong TKF, et al A minimum reporting standard for multiple sequence alignments. NAR Genom Bioinform. 2020:2:lqaa024. 10.1093/NARGAB/LQAA024. 33575581 PMC7671350

[evag061-B50] Yang Z . PAML 4: phylogenetic analysis by maximum likelihood. Mol Biol Evol. 2007:24:1586–1591. 10.1093/MOLBEV/MSM088. 17483113

[evag061-B51] Zhang J . Patterns and evolutionary consequences of pleiotropy. Annu Rev Ecol Evol Syst. 2023:54:1–19. 10.1146/ANNUREV-ECOLSYS-022323-083451/CITE/REFWORKS. 39473988 PMC11521367

[evag061-B52] Zhou J, Yu W, Hardin PE. CLOCKWORK ORANGE enhances PERIOD mediated rhythms in transcriptional repression by antagonizing E-box binding by CLOCK-CYCLE. PLoS Genet. 2016:12:e1006430. 10.1371/JOURNAL.PGEN.1006430. 27814361 PMC5096704

